# Does gender influence learning, perceptions and retention in regional anatomy dissection courses?

**DOI:** 10.1007/s12565-025-00834-5

**Published:** 2025-04-05

**Authors:** Veronica Antipova, Martin Siwetz, Franz A. Fellner, Simone Manhal, Julian F. Niedermair, Benjamin Ondruschka, Amélie J. Poilliot, Monika Wimmer-Röll, Andreas Wree, Niels Hammer

**Affiliations:** 1 Division of Macroscopic and Clinical Anatomy, Gottfried Schatz Research Center, Medical University of Graz, Auenbruggerplatz 25, 8036 Graz, Austria; 2 Central Radiology Institute, Johannes Kepler University Hospital, Linz, Austria; 3 Division of Virtual Morphology, Institute of Anatomy and Cell Biology, Johannes Kepler University, Linz, Austria; 4 Office of the Vice-Rector for Studies and Teaching, Medical University of Graz, Graz, Austria; 5 Institute of Legal Medicine, University Medical Center Hamburg-Eppendorf, Hamburg, Germany; 6 Anatomical Institute, University of Basel, Basel, Switzerland; 7 Institute of Anatomy and Cell Biology, Johannes Kepler University, Linz, Austria; 8 Institute of Anatomy, Rostock University Medical Center, Rostock, Germany; 9 Department of Orthopedic and Trauma Surgery, University of Leipzig, Leipzig, Germany; 10 Division of Biomechatronics, Fraunhofer Institute for Machine Tools and Forming Technology (IWU), Chemnitz and Dresden, Germany

**Keywords:** Embalming, Ethanol-glycerin, Gender difference, Objective structured practical examination, Regional anatomy dissection course, Thiel, Undergraduate medical education

## Abstract

Medical educators should understand the differences within the medical student population, including gender. Research on gender effects on learning and skill acquisition has yielded contradictory results, particularly in anatomy learning among undergraduate medical students. While various dissection course settings have been evaluated, gender-specific differences have largely been overlooked. This study examined gender differences in several aspects of anatomy education. First, the ability of undergraduate medical students to identify anatomical structures was assessed to determine gender differences in learning gains. Second, learning outcomes were compared between a 3-month and a 1-month regional anatomy course, with attention to gender. Third, knowledge retention was evaluated at 6- and 12-month follow-ups. Finally, student perceptions of different embalming methods for anatomy learning were assessed with a focus on gender. Minor differences were observed between genders across different regions in learning gains. Scores from the 3-month course were higher than those from the 1-month course, regardless of gender, particularly for the neck, thorax, and abdomen. A significant knowledge gain was noted in abdominal anatomy at the 6- and 12-month follow-up: scores improved by 56% in females and by 68% in males. The comparison of Thiel- versus ethanol-based embalming revealed gender differences regarding tissue pliability and the utility of tissues for achieving learning objectives. In conclusion, subtle differences appear to exist between female and male students in terms of perceptions and learning outcomes in gross anatomy dissection courses. These findings will be useful in developing new curricula for undergraduate medical students, taking into account gender differences in the context of regional anatomy dissection courses.

**Supplementary Information:**

The online version contains supplementary material available at 10.1007/s12565-025-00834-5.

## Introduction

Anatomy forms an indispensable pillar of medical education (Hurwitz et al. 2013; O'Keeffe et al. 2019; Shojaei et al. 2022; Rehman et al. 2022; Koop et al. 2021). The anatomy dissection course remains a significant part of human and dental medical training worldwide (Korf et al. 2008; Memon 2018; Estai and Bunt 2016; Eisma et al. 2013).

Anatomical dissection provides an excellent opportunity to study spatial alignment and variability, which cannot solely be captured by textbooks or models (Biasutto et al. 2006; Saltarelli et al. 2014; Balta et al. 2015a). Dissection promotes practical skills, teamwork, self-reflection and independent learning. It helps guide the student to respect humanistic values and thus promotes emotional maturity in the direction of professional, medical behavior (Ali et al. 2015; Larkin and McAndrew 2013; Theoret et al. 2007, Kumar Ghosh and Kumar 2019).

Embalmed human tissue not only forms the basis of gross anatomy training for more than one century (Hammer et al. 2011; Brenner 2014; Drake et al. 2009; Kennel et al. 2018; Rakuša and Kocbek Šaherl 2022), but is also indispensable for postgraduate workshops and for research (Hammer et al. 2015, 2012; Choi et al. 2020; Crosado et al. 2020).

Gender and associated roles have been reported to impact learning and skills acquisition (Garg et al. 2001; Guillot et al. 2007; Thorson et al. 2011). With changing female-to-male student ratios in medicine and dentistry in the last two decades, these differences warrant further evaluation (Mooij et al. 2011; Habumuremyi et al. 2024). Only limited studies so far have dealt with gender differences in student performance in medicine, especially in performance in regional anatomy dissection. Contemporary findings on the effect of gender on learning and skills acquisition (Garg et al. 2001; Guillot et al. 2007; Thorson et al. 2011; Szczepanik et al. 2010; McDonough et al. 2000; Smits et al. 2004; Melovitz-Vasan 2018; Kelly and Dennick 2009) are conflicting and warrant further investigation in the field of gross anatomy.

The role of gender in anatomical dissection courses was not previously focused on in our studies (Antipova et al. 2023, 2024a, b; Niedermair et al. 2023). Given the evolving student demographics and the potential impact of gender on learning outcomes, this study seeks to address a previously unexplored aspect of our research by focusing on gender-specific differences across various settings of the anatomical dissection course. Additionally, we would like to point out that our hypotheses were qualitative, not quantitative, i.e., we made no estimation on the extent of potential difference.

The primary aim of this study was to investigate gender differences among undergraduate medical students in terms of learning gains and their ability to identify anatomical structures. We hypothesized that these parameters would be influenced by gender.

A second aim was to evaluate gender differences in student learning outcomes when comparing a 3-month and a 1-month regional anatomy course setting. We hypothesized that the ability to identify anatomical structures was superior if course contents were delivered over a longer teaching period and would differ between genders.

The third aim of this study was to examine gender differences in knowledge retention among undergraduate medical students at 6- and 12-month follow-ups. It was hypothesized that gender differences would exist in knowledge retention, defined as the ability to identify anatomical structures.

A fourth aim was to assess gender differences in student performance and perceptions regarding two embalming techniques: Thiel and ethanol-glycerin embalming. It was hypothesized that examination scores and student perceptions of tissue suitability to achieve learning objectives would differ between genders.

This study analyzed the above-mentioned aims regarding potential gender differences using objective structured practical examinations (OSPE, “tag examinations”) and structured surveys.

## Materials and methods

A binary approach was employed, focusing solely on the female and male genders. Inclusion of other gender identities was not possible due to the very limited number of students identifying outside this binary, making reliable statistical analysis unattainable. Additionally, the vast majority (over 95%) of Austrian medical students are reflected as identifying as either female or male (own unpublished results).

### Course details on the anatomy curricula at the Johannes Kepler University Linz and the Medical University of Graz

Gross anatomy is a first- and second-year subject taught to undergraduate medical students at both the Johannes Kepler University Linz (JKU) and the Medical University of Graz (MUG). Anatomy teaching is taught dissection-based with human tissues bequeathed to the Division of Macroscopic and Clinical Anatomy of the MUG as a part of an ongoing body donation program and in accordance with the Austrian legislation concerning body donations (https://anatomie.medunigraz.at/en/body-donation). The bodies used for the regional anatomy dissection course were embalmed with ethanol-glycerin according to (Hammer et al. 2012), or with the ‘2002’ Thiel embalming method (Thiel 2002).

Both course settings are taught hands-on at the same anatomy facility in Graz and by experienced MUG anatomists. This practice is based on a memorandum of understanding and supporting agreement between the MUG and the JKU.

For the 3-month dissection course, the framing curriculum is designed as follows: anatomy is taught in the first three semesters as a part of different modules and tracks. In the first semester, 20 lectures are delivered on gross anatomy, focusing on the general anatomy of the skeletal system and osseous structures, accompanied by individual self-study units and followed by an oral examination. In the second semester, two systematic anatomy modules are taught on the musculoskeletal and nervous system. Both modules comprise gross anatomy, histology, physiology and biophysics, with 46 h and 41 h of lectures, and 36 h and 20 h of dissection for the musculoskeletal and nervous system modules, respectively. The 3-month dissection course begins with the third semester and lasts three months. More detail on the undergraduate curriculum framing the 3-month dissection course is found in (Antipova et al. 2023, 2024a).

For the 1-month dissection course setting, the curriculum was designed as follows: teaching units held to small groups were offered using plastinates and models, accompanied by lectures on general and systematic anatomy as well as histology over the first semester. Students in their first semester were taught 18 contact hours in general anatomy, comprising of lectures on the musculoskeletal and cardiovascular system, head and neck, trunk, upper and lower limbs and the peripheral nervous system with a clear focus on the musculoskeletal system. Additionally, nine lectures and 39 seminars were held in preparation for the regional anatomy dissection course. The 1-month dissection course formed at the beginning of the second semester and had a duration of four full weeks. Intensive advanced anatomy classes, including a course in virtual anatomy, took place in this cohort after the dissection course in the 3rd and 4th semesters. More detail on the undergraduate curriculum framing the 1-month dissection course is presented in (Niedermair et al. 2023; Antipova et al. 2023, 2024a).

Both the 1- and 3-month dissection courses are highly similar in terms of teaching content, accompanying lecture, times of hands-on dissection (60 units of 45 minutes each) and examination mode; and both are based on the same course manual (Antipova et al. 2023, 2024a). Both courses are designed to be self-standing. In line with the curricula of both universities, hands-on dissection comprised 3 units per day five times per week for four weeks for the JKU cohort, and 3 units per day held typically twice per week for three months for the MUG groups, respectively. Participation in the anatomy course was mandatory for all students; minimum course attendance was 85% as per the rules of the MUG curriculum. The key difference had been that the courses for the 1-month cohort were delivered five times per week for four weeks, and typically twice per week for three months, respectively.

Both the 1-month and the 3-month dissection courses were accompanied by 94 online regional anatomy lectures. Twelve to eighteen students were allocated per body, and divided into two groups. Each student was allocated one or several anatomical regions for guided dissection supported by experienced gross anatomists and student demonstrators. The ratio of students to demonstrators to academic teachers averaged 32: 2: 1. For all four aims of this study, OSPE examinations were conducted following the dissection of the respective topographical regions before the students’ respective oral examinations. All participants gave their written and informed consent prior to their voluntary participation. Ethical approval was obtained from the ethical committee of the Medical University of Graz, Austria (33-500 ex 20/21).

### Data acquisition

For the OSPE, six to ten numbered tags were marked in the prosections of regions and placed prominently to clearly identify specific anatomical structures. The same structures were tagged in ethanol-glycerin- and Thiel-embalmed prosections in the same way (Antipova et al. 2023; Niedermair et al. 2023). One multiple-choice question was asked per tag, requiring the participants to select only the most suitable out of five possible answers (Antipova et al. 2023). Students were allotted one minute per question, while neither manipulation of the numbered tags nor touching of the structures was allowed to keep the test setting standardized. Only questions on topics that were specified in exams in the gross anatomy dissection course were included. Structure and item selection for the multiple-choice questions were performed by experienced anatomists and a psychologist. The students were assigned randomly to the embalming method. Throughout the whole dissection course and self-study all students had access to both embalming types.

Based on the absolute numbers of correct responses, a percentage was calculated for each region the students participated in. To negate observer bias, one set of prosections was prepared based on ethanol-glycerin and another based on Thiel embalming for each anatomical region in line with the embalming methods offered in the regional anatomy course.

Following the last OSPE, the students were surveyed on their opinion regarding the suitability of ethanol-glycerin and Thiel embalming concerning tissue preservation, colorfastness, tissue pliability and perceptions on tissue suitability to achieve the anatomy learning objectives (Antipova et al. 2023). For this purpose, a five-point Likert scale was adopted (1 = most suitable/strongly agree, 5 = most unsuitable/strongly disagree) (Likert 1932). This survey was conducted without the students knowing the results of the OSPE (Antipova et al. 2023, 2024a).

For the third aim of the given study, the first OSPE examinations were conducted immediately following the completion of the dissection of each anatomical region prior to the respective oral examinations (pre exam, pre scores). The follow-up examinations were carried out 6- and 12-month (6-month and 12-month scores) following the pre exam (Antipova et al. 2024b).

Between both follow-up examinations, lectures on virtual anatomy, pathology and further seminars were held in Linz (Niedermair et al. 2023; Antipova et al. 2024b).

### Statistical evaluation

Data on the examination scores as well as personal data were analyzed using Prism version 9 (GraphPad Software Inc., La Jolla, CA, USA) and Microsoft Excel version 16.49 (Microsoft Corp., Armonk, NY, USA). Normal distribution was assessed using the D’Agostino and Pearson test. A Kruskal–Wallis test with post-hoc correction (Dunn’s test) was used for between-group comparison of the pre- and post-examination assessments, and separated for ethanol-glycerin and Thiel embalming. A Kruskal–Wallis test without post-hoc correction (uncorrected Dunn’s test) was used for the comparison of the between-gender difference in the relative learning gain. *P* values ≤ 0.05 were considered statistically significant.

## Results

### Male gender scored higher for abdomen and lower extremity

**Fig. 1:**
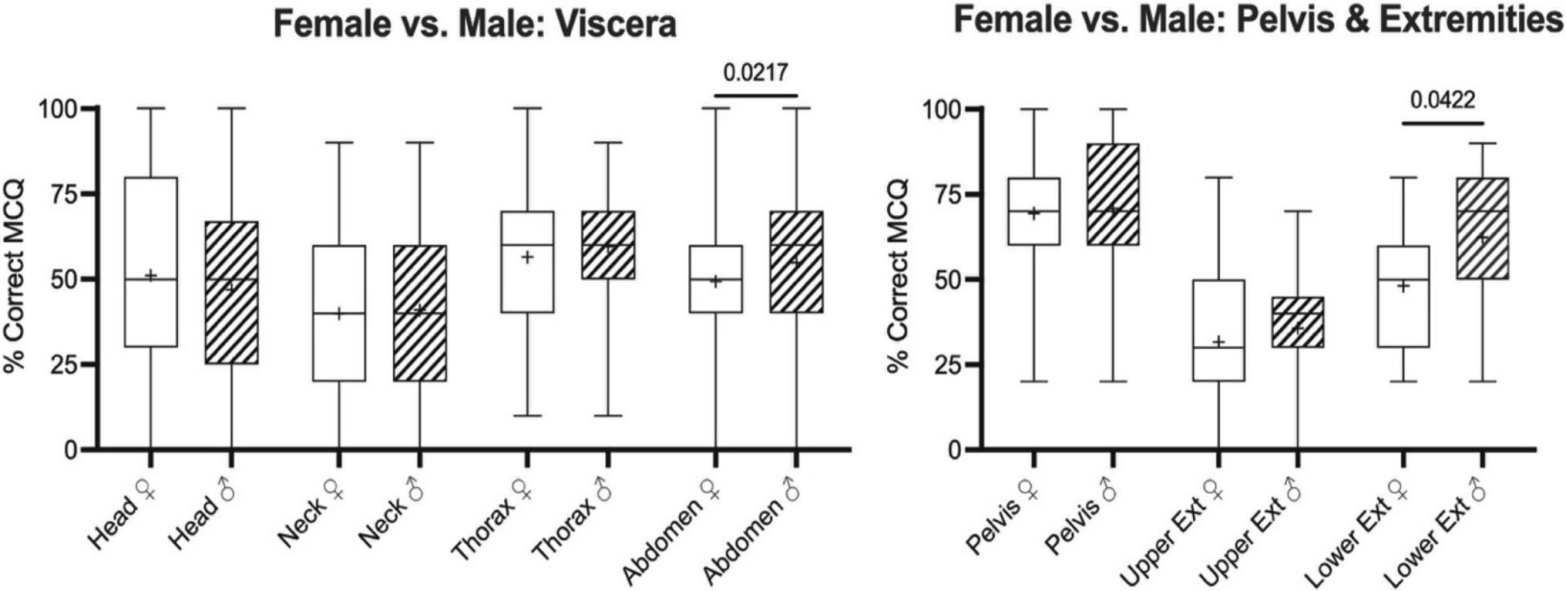
Box plots comparing the scores of the objective structured practical examinations separated for both genders. The boxes indicate the 25th percentile, median and 75th percentile, whiskers the minima and maxima. Crosses depict the mean value. Higher scores were achieved for males when compared to females in the abdomen and lower extremity (Ext) regions

Broad variation was observed when comparing the OSPE scores between female (*n* = 244) and male (*n* = 212) gender (total of 477 students, 21 not given), independent of the embalming method used. Males achieved higher scores in the abdomen (49 ± 20% vs. 55 ± 20%; *p* = 0.022) and lower extremity regions (48 ± 18% vs. 62 ± 19%; *p* = 0.042) when compared to females (Fig. 1). No difference between genders was observed for the head (51.11 ± 31.05% vs. 46.99 ± 27.30%), neck (40 ± 22% vs. 41 ± 22%) and thorax regions (56 ± 20% vs. 60 ± 19%; all *p* ≥ *0.529*). Similarly, no difference was observed for the pelvis (69 ± 16% vs. 71 ± 21%) and upper extremity regions (32 ± 18% vs. 36 ± 18%; all *p* ≥ 0.999).

### Longer course durations yielded superior learning gain for both female and male gender and in most regions

**Fig. 2:**
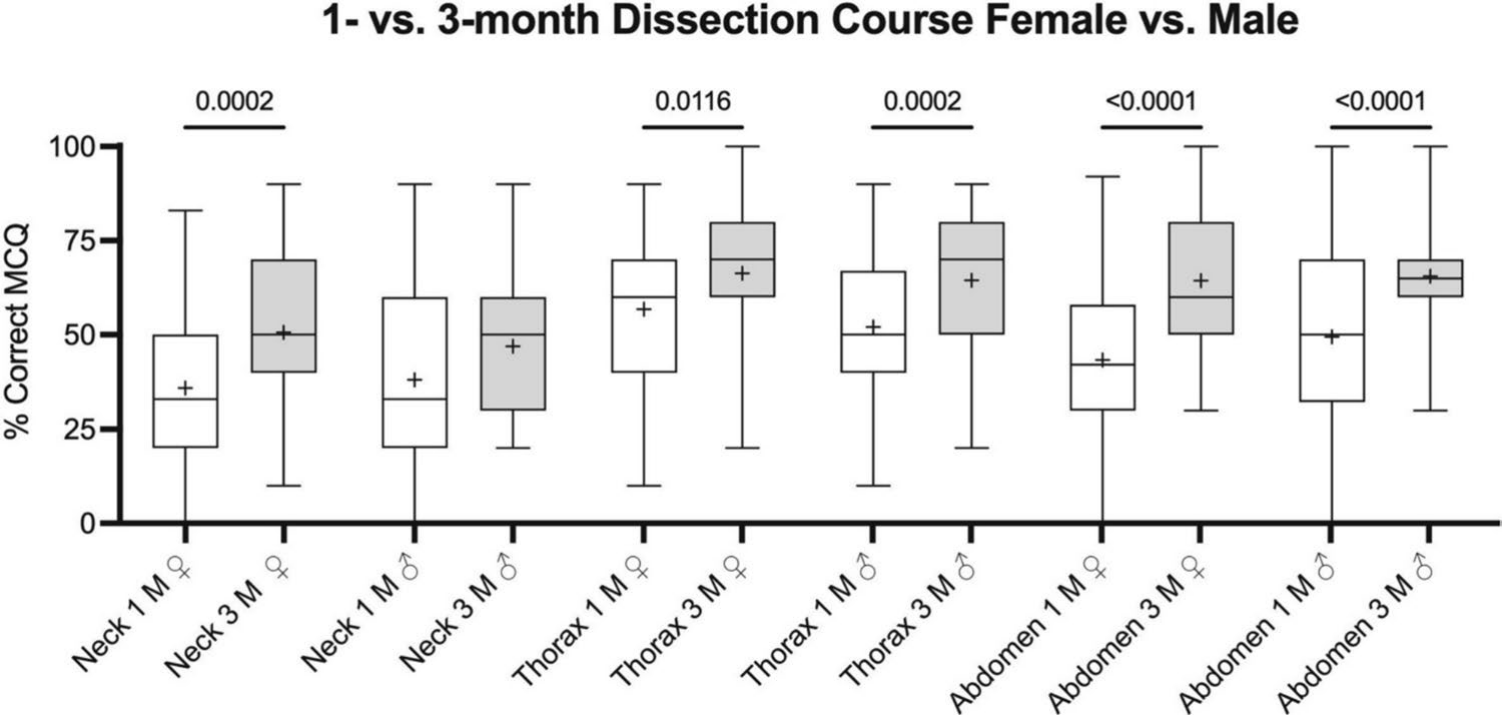
Box plots comparing the examination scores of the 1- (1 M) and 3-month (3 M) dissection course settings for the neck, thorax and abdomen regions, separated for female and male subgroups. The boxes indicate the 25th percentile, median and 75th percentile, whiskers the minima and maxima. Crosses depict the mean value. Significantly higher scores were consistently observed in both genders for the 3-month dissection course setting, except for the neck region in the male student cohort

Comparison of student learning outcomes following a 1-month versus a 3-month regional anatomy course under otherwise identical conditions demonstrated superior learning gain for both genders and most anatomical regions (total of 466 students, 241 females, 209 males, 16 not given). Females yielded higher 3-month scores for the neck (36 ± 21% vs. 51 ± 20%, *p* = *0.0002*), thorax (57 ± 20% vs. 66 ± 17%, *p* = *0.0116*) and abdomen regions (43 ± 19% vs. 64 ± 16%, *p* < *0.0001*). Male yielded higher scores for the 3-month scores of the thorax (52 ± 20% vs. 65 ± 16%, *p* = *0.0002*) and abdomen regions (49 ± 21% vs. 66 ± 14%, *p* < *0.0001*) (Fig. 2).

### Knowledge retention for the abdomen region was irrespective of gender

**Fig. 3:**
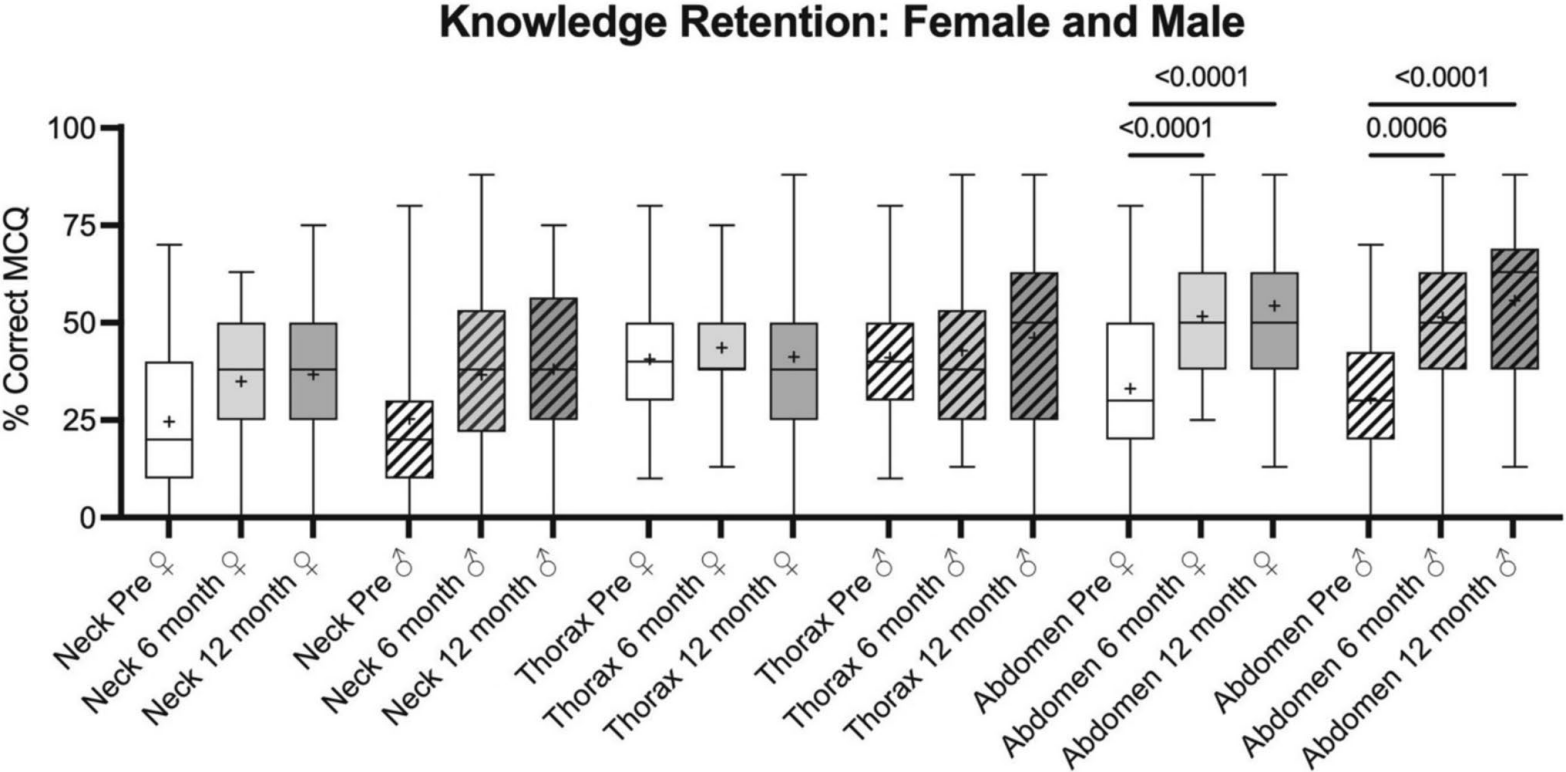
Box plots comparing the Pre, 6- and 12-month examination scores of the neck, thorax and abdomen regions separated for female and male participants. The boxes indicate the 25th percentile, median and 75th percentile, whiskers the minima and maxima. Crosses depict the mean value. Significant change in examination scores was primarily found for the abdominal region. Comparison of between-gender differences in the relative learning gain (i.e., pre vs. 6-month, pre vs. 12-month, 6-month vs. 12-month) yielded no difference

When assessing knowledge retention of students completing a 1-month regional anatomy dissection course in a 6- and 12-month follow-up, a difference was observed for the abdominal region pre-examination vs. 6- and 12-month follow-up scores for female (33.11 ± 20.94% vs. 51.67 ± 14.29% vs. 54.37 ± 17.99%, *p* < *0.0001*, *n* = 63; *n* = 52; *n* = 51) and male genders (30.53 ± 17.85% vs. 51.35 ± 14.29% vs. 55.68 ± 16.74%, *p* ≤ *0.0006*, *n* = 41; *n* = 38; *n* = 37), i.e. scores improved by 56% in the female and by 68% in the male gender. No significant difference was observed between the gender (Fig. 3). Further comparison of learning retention related to the interventions (i.e., pre vs. 6-month, pre vs. 12-month, 6-month vs. 12-month) demonstrated no difference between genders (*p* ≥ 0.5757).

### Females rated Thiel tissue more pliable, both genders found ethanol-glycerin preservation better to achieve learning objectives

**Fig. 4:**
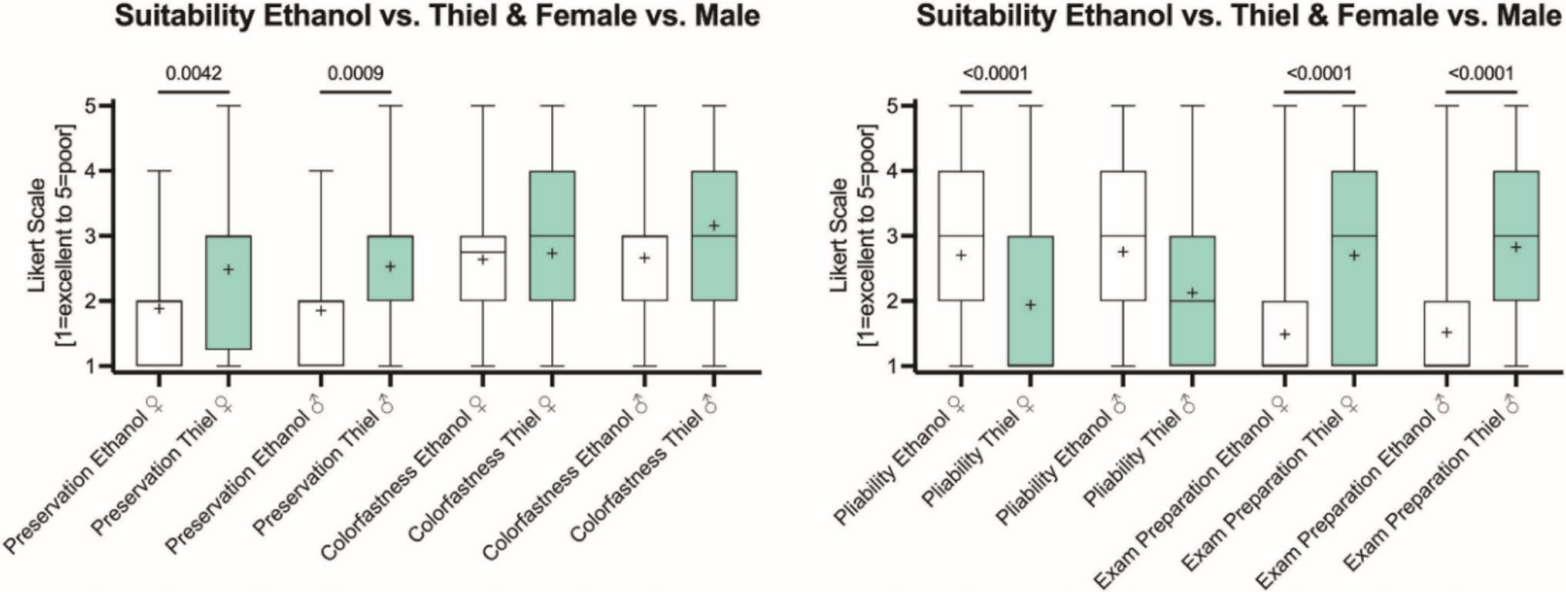
Box plots on the assessment of Thiel and ethanol-glycerin (Ethanol) embalming based on Likert ratings. The boxes indicate the 25th percentile, median and 75th percentile, whiskers the minima and maxima. Crosses depict the mean value. Female and male students likewise found ethanol-glycerin embalming to provide better tissue preservation and to achieve the anatomy learning objectives. Only females found Thiel-embalmed tissues to be more pliable than ethanol-glycerin-embalmed tissues (*n* ≥ 72)

Further comparison yielded no gender difference regarding the suitability of Thiel or ethanol-glycerin embalming for their learning (Fig. 4; *p* ≥ *0.061*) except for tissue pliability, which females rated to be superior in Thiel embalming (244 females, 212 males, 21 not given). Female and male students likewise found ethanol-glycerin embalming to provide better tissue preservation and achieve anatomy learning objectives.

## Discussion

The number of female medical students has steadily increased over the past few decades, now outnumbering male students in medical schools (Burton and Wong 2004; Yusuf and Elfaki 2022; Blanch et al. 2008; Yaman et al. 2023). To develop training and curricula that produce the best possible physicians, and to narrow the gender gap while making education more inclusive, medical educators must understand the gender differences within the medical student population (Blanch et al. 2008; Habumuremyi et al. 2024). Therefore, researchers and educators are interested in examining the impact of gender on the academic performance of medical students (Yusuf and Elfaki 2022; Blanch et al. 2008; Mooij et al. 2011). Gender differences in academic performance among male and female medical students were addressed in numerous studies, however with variable and conflicting results (Yusuf and Elfaki 2022; Burton and Wong 2004; Sheard 2009; Bienstock et al. 2002; McDonough et al. 2000). Moreover, gender bias in subjective performance evaluations of medical trainees was highly heterogeneous (Gerull et al. 2019; Meyerson et al. 2017; Sulistio et al. 2019; Brucker et al. 2019; Acuña et al. 2020; Lane et al. 2020; Hoops et al. 2019; Rojek et al. 2019; Yaman et al. 2023).

To our knowledge, no study has so far addressed gender differences in learning outcomes, perceptions, and knowledge retention in undergraduate medical students related to a regional anatomy dissection course.

### Anatomy dissection and identification involves spatial ability skills

Firstly, the learning gain and related ability to identify anatomical structures were studied in Thiel- and ethanol-glycerin-embalmed tissues (Antipova et al. 2023, 2024a) of the undergraduate medical students using OSPE (Vishwakarma et al. 2016; Dissabandara et al. 2023) in relation to gender difference. Here only a few differences were found between genders for the different regions. Therefore, these results are partially consistent with our hypothesis that the learning gain and related ability to identify anatomical structures of undergraduate medical students would be influenced by gender.

Males scored higher for the abdomen and lower extremity regions, however, no difference between genders was found for the head, neck and thorax regions, for the pelvis and upper extremity regions (Fig. 1). The explanation seems multifactorial. In addition to learning style, gender and associated roles were reported to impact learning and skills acquisition (Garg et al. 2001; Guillot et al. 2007; Thorson et al. 2011). Spatial ability involves the mental manipulation of objects and the appreciation of the spatial relations between objects (Linn and Petersen 1985; Pellegrino et al. 1984; Wai et al. 2009, Yilmaz, 2009). This is important in various fields including STEM (science, technology, engineering, and mathematics) education (Wai et al. 2009), engineering (Contero et al. 2006; Roca-González 2017), and medical disciplines (Hegarty et al. 2010) including surgery (Beattie et al. 2022; Rogister et al. 2022) and radiology (Birchall 2015; Nilsson et al. 2007). Understanding the importance of spatial ability and its implications in teaching anatomy is essential, as anatomical structures are related to each other in a 3-dimensional space (Yousuf et al. 2024; Bogomolova et al. 2020; Silén et al. 2022; Pettersson et al. 2023). Anatomy students must manipulate and rotate anatomical specimens to improve their ability to visualize the spatial relationships between various anatomical structures in different planes. Consequently, spatial ability is crucial for learning anatomy (Roach et al. 2021; Sarilita et al. 2022; Yousuf et al. 2024; Bogomolova et al. 2020; Ritchie et al. 2023; Wang and McWatt 2023).

There is evidence of a strong relationship between spatial abilities and anatomical competence, and males scored higher than females in visuospatial representation and mental rotation (Garg et al. 2001; Guillot et al. 2007). Males also reportedly tend to perform better in tasks within a simulated environment (Thorson et al. 2011). However, the reasons remain unknown in detail to date. This finding could be partially explained by males playing more action video games which enhance their mental rotation ability (Podlogar and Podlesek 2022; Spence and Feng 2010). Geary has suggested that the difference may be due to males’ tendency to orient toward a goal using distant landmarks, whereas women may not use this strategy for comparable goals frequency (Geary 2021). Yuan and colleagues gave an anatomical-physiological explanation to this gender discrepancy postulating that the reason why females performed not so well in large-scale spatial ability was because they were more susceptible to emotions and their parahippocampal gyrus worked less efficiently compared with males (Yuan et al. 2019). It is vital to note that the difference between male and female genders in tasks such as mental rotation, may also disappear depending on several factors (Jansen-Osmann and Heil 2007; Hegarty 2018; Ruthsatz et al. 2019; Alvarez-Vargas et al. 2020; Jost and Jansen 2024). Although there are several factors influencing the learning outcomes, including the social, psychological and neurophysiological factors mentioned above, this may lead to an explanation as to why a gender difference was found with higher scorings for males in the abdomen and lower extremity. However, it is important to note that these differences were limited to these two regions. So, there are factors promoting a better scoring in males, however, these were limited to the abdomen and lower extremity and were not found in other regions such as the thorax. Future research will have to clarify these apparent inconsistencies.

### There is no evidence for gender-different pattern in examination performance for shorter versus longer course durations

The second aim of this study was to compare student learning outcomes of a 3-month versus a 1-month anatomy course in relation to gender. Recently, we showed irrespective of gender that 3-month course exposure resulted in significantly higher OSPE scores for the neck, thorax, and abdomen and the course exposure over a more extended period like 3 months appears to be highly beneficial (Antipova et al. 2024a).

Undergraduate medical students in the 3-month dissection course scored higher across all regions, likely due to the increased frequency of exposure to anatomical structures (Antipova et al. 2024a). Studies suggest that pattern recognition improves with experience, while stress, particularly in shorter courses, can negatively impact student performance and learning outcomes (Schlachta et al. 2015; Tyng et al. 2017; Rowe and Fitness 2018). Compared to the 1-month course, the female gender yielded higher 3-month scores for the neck, and both the female and male gender showed higher scores for the thorax and abdomen regions (Fig. 2). These results are only partially consistent with our hypothesis that the ability to identify anatomical structures was superior if course contents were delivered over a longer teaching period and would differ between genders.

Abdellatif found a positive correlation between dissection time and anatomy knowledge (Abdellatif 2020), while Nwachukwu and colleagues noted that reduced laboratory hours negatively affect performance (Nwachukwu et al. 2015). Similarly, Insausti and colleagues reported improved performance with minimal self-directed learning (Insausti et al. 2022), and Holla and colleagues linked longer curricula with better clinical understanding, though these studies vary in content and are difficult to compare (Holla et al. 2009).

### Knowledge retention is similar between female and male gender with specific improvements for the abdomen region

The third objective was to assess knowledge retention in male and female medical students who completed a 1-month regional anatomy dissection course, with follow-ups at 6 and 12 months (Fig. 3). Significant score improvements were observed only in the abdominal region, with increases of 56% in females and 68% in males, while no differences were found in the neck and thorax regions. These results suggest that “spaced learning” leads to better retention than “massed learning” (Smolen et al. 2016; Cepeda et al. 2008), and additional teaching interventions, such as virtual anatomy and plastic models, further enhance retention in the abdominal region (Niedermair et al. 2023). The findings challenge the hypothesis that short courses result in poor retention, highlighting the importance of course duration, spaced instruction, and supplementary educational methods in long-term knowledge retention (Kerfoot et al. 2007; Smolen et al. 2016). The fact that these findings are similar for both female and male genders concerning the intellectual mechanisms relating to knowledge increase and “spaced” versus “massed learning”. These results reject our hypothesis that gender differences would exist in knowledge retention, defined as the ability to identify anatomical structures.

### Gender difference in perceptions on tissue qualities may impact the learning outcomes

Snelling and colleagues investigated how attitudes toward dissection vary by gender and ethnicity, including students' reactions and concerns regarding the dissecting room, the coping strategies they use, and effective methods for teaching anatomy to medical and dental students (Snelling et al. 2003). Females consistently expressed greater concern about the physical aspects of dissection, while there were fewer gender differences in emotional responses. Gender differences so far have mostly been found in the domains of anxiety and the mode of coping (Hancock et al. 2004). Males were more motivated by the dissection experience than females, and as the level of anatomical knowledge increases, motivational scores also increase (Abdel Meguid and Khalil 2017). Although the majority of medical students perceive initially that dissection was a positive life event (O'Carroll et al. 2002), increased stress rates were reported in female students to the dissection practicals (Abu-Hijleh et al. 1997; Snelling et al. 2003; Sándor et al. 2015). The fact that women react more sensitively than men to the dissection-room experiences may have long-term consequences in a central issue in medicine today, the feminization of the profession.

Vice versa, Yates and James found male medical students were at a greater risk of struggling than females (Yates and James 2006). Female students are more likely to use effective study methods, better manage their time and seek formative feedback (Sinclair and Cleland 2007; Marrs and Sigler 2011). Habumuremyi and colleagues found no difference between genders in student performance in anatomy (Habumuremyi et al. 2024). Melovitz-Vasan and colleagues also found no difference between genders in anatomy. Gender poses no limitation to medical student performance, irrespective of the type of examination format (Melovitz-Vasan 2018).

Concerning gender differences regarding the suitability of Thiel or ethanol-glycerin, no difference was found except for tissue pliability rated higher for Thiel embalming by females (Fig. 4). Both female and male students likewise found ethanol-glycerin embalming to provide better tissue preservation and to achieve the anatomy learning objectives. On one hand, these results partially fit with our hypothesis that examination scores and student perceptions of tissue suitability to achieve learning objectives would differ between genders. Previous studies, including those in the same cohorts, show that embalming affects learning outcomes (Antipova et al. 2023). This study compared ethanol-glycerin and Thiel-based methods. Ethanol stiffens and bleaches tissues, aiding fine structural identification, while Thiel preserves lifelike properties but may require a gamified approach to reveal details (Cale and McNulty 2025). Thus, embalming may influence gender-related learning differences, which may have also gone in line with gender-different evaluations of tissue properties as outlined in Fig. 4. On the other hand, these results agree with the results of Balta and colleagues, who compared formaldehyde with Thiel embalming in an undergraduate and postgraduate teaching environment (Balta et al. 2015b). The same undergraduate group also found that Thiel-embalmed tissues were more advantageous for the identification of limb structures (Balta et al. 2015b). The bleaching and induration related to ethanol-glycerin presumably make it easier for students to identify relevant structures, and facilitate dissection (Hammer et al. 2011, 2012, 2015).

Therefore, ethanol-glycerin appears to be a more suitable method of fixation for viscera in an undergraduate setting when compared to Thiel (Antipova et al. 2023). In consequence, students’ ideas on tissue suitability for their learning align with existing research irrespective of gender. As a result, students’ ideas about the suitability of tissue for their learning, regardless of gender, are consistent with existing research.

## Future directions

Further study is necessary to better elucidate the factors influencing the impact of gender on academic performance of undergraduate medical students in regional anatomy. In addition, it may be helpful to evaluate whether similar results to those presented here are applicable to other standard embalming techniques—beyond ethanol-glycerin and Thiel fixation and in a more heterogeneous group, including more than only female and male genders. Furthermore, based on the differences in subjective perceptions of different embalming methods, it may be assessed whether these perceptions do influence the learning outcomes.

## Limitations of the study

This study has a number of limitations. First, using this binary approach separating females and males only, it can be expected that this study falls short of representing the full picture of gender allocation and related learning outcomes. Further studies are needed to consider gender differences that extend beyond females and males. It must be mentioned, however, that a vast majority (i.e., more than 95%) of the Austrian medical students remain reflecting themselves as being of female or male gender. Second, only a limited number of undergraduate medical students was assessed. Third, the OSPE has certain limitations as a method of assessing student performance that are beyond the scope of this publication. Furthermore, the present results are limited to undergraduate medical education and it remains unclear whether the results are applicable beyond Austrian medical education.

## Conclusions

Few differences were found between genders in learning gains and the ability to identify anatomical structures in Thiel- and ethanol-glycerin-embalmed tissues using OSPE. Except for the better rating of tissue pliability by females in Thiel embalming, no significant differences were observed between genders regarding the suitability of either embalming method. Performance in hand conditions was similar across genders, though males demonstrated superior processing of haptic information. Ethanol-glycerin embalming was preferred by both genders for better tissue preservation and achieving learning objectives. In a 1-month regional anatomy course, significant improvements were noted in abdominal region scores, with increases of 56% for females and 68% for males at 6- and 12-month follow-ups. These results suggest that knowledge retention is influenced by course duration, spaced instruction, and additional teaching interventions irrespective of female or male gender. As the ratio of female students has increased, these findings may guide the development of curricula which consider gender differences in anatomy education.

## Supplementary Information

Below is the link to the electronic supplementary material.

Supplementary file1 (PDF 136 kb)

## Data Availability

Data collected during this study are available from the corresponding author upon reasonable request.
