# Supplementary material for: Does gender influence learning, perceptions and retention in regional anatomy dissection courses?
Source: Anat Sci Int. 2025 Apr 5;101(1):61–72. doi: 10.1007/s12565-025-00834-5 (PMC12804274; doi:10.1007/s12565-025-00834-5)
Supplement: Supplementary file 1 — Supplementary file1 (PDF 136 kb) [file 12565_2025_834_MOESM1_ESM.pdf]

OBJECTIVE STRUCTURED PRACTICAL EXAMINATIONS (‘TAG EXAMS’)

Abdomen

1. **Which of the following structures is labeled with number 1?**

Hepatoduodenal ligament

(A) The hepatoduodenal ligament.

(B) The gastroduodenal ligament.

(C) The greater omentum.

(D) The round ligament of the liver.

(E) The gastrocolic ligament.

2. **Which of the following structures is labeled with number 2?**

Superior mesenteric artery

(A) The coeliac trunk.

(B) The superior mesenteric artery.

(C) The inferior mesenteric vein.

(D) The superior pancreatoduodenal artery.

(E) The middle colic artery.

3. **Which of the following structures is labeled with number 3?**

Umbilical artery

(A) The umbilical artery.

(B) The umbilical vein.

(C) The median umbilical fold.

(D) The lateral umbilical fold.

(E) The inferior epigastric artery.

4. Which of the following statements holds true for the structure labeled with number 4?

Right colic artery

(A) The labeled structure is a branch of the inferior mesenteric artery.

(B) The labeled structure anastomoses with the ovarian artery.

(C) The labeled structure is responsible for the vascular supply of the duodenum.

(D) The labeled structure forms a common origin with the ileocolic artery.

(E) When the labeled structure is obliterated, the descending colon becomes ischemic.

5. Which of the following structures is labeled with number 5?

Gall bladder

(A) The caudate lobe of the liver.

(B) The bile duct.

(C) The common hepatic duct.

(D) The cystic duct.

(E) The gall bladder.

6. Which of the following statements holds true for the structure labeled with number 6?

Umbilical vein

(A) The labeled structure is located in the median umbilical fold.

(B) The labeled structure gives off branches to the porta hepatis.

(C) The labeled structure is located in the hepatoduodenal ligament.

(D) The labeled structure enters the abdominal aorta.

(E) The labeled structure continues into the round ligament of the liver.

7. Which of the following statements holds true for the structure labeled with number 7?

<u>Obliquus internus muscle</u>

(A) The labeled structure does not insert into the aponeurosis of the rectus abdominis muscle.

(B) The labeled structure forms an anterior and posterior sheet below the arcuate line.

(C) The labeled structure forms the semilunar line.

(D) The labeled structure is exclusively innervated by the lumbar nerves.

(E) The labeled structure originates on the inguinal ligament.

8. Which of the following statements holds true for the structure labeled with number 8?

<u>Rectus abdominis muscle</u>

(A) The labeled structure can have up to 8 muscle bellies on each side.

(B) The labeled structure is not involved in the abdominal press.

(C) The labeled structure has a primary extensor function in the lumbar spine.

(D) The labeled structure is connected to the rectus sheath via tendinous intersections.

(E) The labeled structure is exclusively innervated by the 11th and 12th intercostal nerves.

9. Which of the following statements holds true for the structure labeled with number 9?

<u>Spleen</u>

(A) The labeled structure plays a role in the immune system.

(B) The labeled structure has a small and a large curvature.

(C) The labeled structure has a thick fibrous capsule.

(D) The labeled structure is surrounded by paranephric fat.

(E) The labeled structure is supplied by the gastro-omental artery.

10. Which of the following statements holds true for the structure labeled with number 10?

<u>Superior epigastric artery</u>

(A) The labeled structure is part of the collateral circulation in portal hypertension.

(B) The labeled structure anastomoses with the internal thoracic artery.

(C) The labeled structure takes a course anterior to the rectus abdominis muscle in the rectus sheath.

(D) The labeled structure anastomoses with the same structure on the opposite side.

(E) The labeled structure is called superficial epigastric artery.

Thorax

1. Which of the following statements holds true for the structure labeled with number 1?

Left coronary artery

(A) The labeled structure is responsible for the vascular supply of the left and right ventricle.

(B) The labeled structure gives off the posterior interventricular branch.

(C) The labeled structure originates off the left aortic sinus behind the mount of the aortic valve.

(D) The labeled structure takes a course between the arterial conus and the right auricle.

(E) The labeled structure takes on the posterior interventricular branch.

2. Which of the following structures is labeled with number 2?

Anterior papillary muscle

(A) Anterior papillary muscle

(B) Left papillary muscle

(C) Right papillary muscle

(D) Posterior papillary muscle

(E) Septal papillary muscle

3. Which of the following statements for the structure labeled with number 3 is incorrect?

Pulmonary artery

(A) The labeled structure is a pulmonal artery.

(B) The labeled structure is a pulmonal vein.

(C) The labeled structure is part of the public vessels of the lung.

(D) The labeled structure has a middle tunica.

(E) The labeled structure is a terminal vessel.

4. Which of the following statements holds true for the structure labeled with number 4?

Oblique fissure

(A) The labeled structure is the horizontal fissure of the lung.

(B) The labeled structure separates the lung into a superior and middle lobe.

(C) The labeled structure is the so-called transverse fissure of the lung.

(D) The labeled structure separates the lung into a middle and inferior lobe.

(E) The labeled structure separates the lung into a superior, middle and inferior lobe.

5. Which of the following statements holds true for the structure labeled with number 5?

Internal thoracic artery

(A) The labeled structure is a branch of the thoracic aorta.

(B) The labeled structure gives off the pericardiophrenic artery.

(C) The labeled structure takes a course accompanied by the phrenic nerve.

(D) The labeled structure is a branch of the vertebral artery.

(E) The labeled structure is a branch of the thyrocervical trunk.

6. Which of the following statements for the structure labeled with number 6 is incorrect?

<u>Thoracic duct</u>

(A) The labeled structure is responsible for the transport of liquids with a high percentage of lipids.

(B) The labeled structure enters the right venous angle.

(C) The labeled structure has its origin in the cisterna chyli.

(D) The labeled structure is responsible for the transport of lymph.

(E) The labeled structure passes the diaphragm in the aortic hiatus.

7. Which of the following statements holds true for the structure labeled with number 7?

<u>Sympathetic trunk</u>

(A) The labeled structure enters the left venous angle.

(B) The labeled structure gives off the sacral splanchnic nerves

(C) The labeled structure is directly connected with the impar ganglion.

(D) The labeled structure is composed of 22-23 paravertebral ganglia.

(E) The labeled structure is composed of 50 autonomous ganglia.

8. Which of the following statements holds true for the structure labeled with number 8?

Pericardiacophrenic artery

(A) The labeled structure anastomoses with the inferior phrenic artery.

(B) The labeled structure is a branch of the superficial thoracic vein.

(C) The labeled structure is responsible for the innervation of the diaphragm.

(D) The labeled structure is accompanied by the vagus nerve.

(**E) The labeled structure anastomoses with the superior phrenic and musculophrenic arteries**.

9. Which of the following statements holds true for the structure labeled with number 9?

Ligamentum arteriosum

(A) The left phrenic nerve takes a course around the labeled structure.

(B) The right vagus nerve takes a course around the labeled structure.

(C) The right recurrent laryngeal nerve takes a course around the labeled structure.

(D) The labeled structure is a thread of connective tissue between the aorta and the pulmonal trunk.

(E) The labeled structure is a remnant of the venous duct.

10. Which of the following statements holds true for the structure labeled with number 10?

<u>Left recurrent laryngeal nerve</u>

(A) The labeled structure is a branch of the phrenic nerve.

(B) The labeled structure is a branch of the vagus nerve.

(C) The labeled structure is a branch of the sympathetic trunk.

(D) The labeled structure is a unilaterally occurring branch of the vagus nerve.

(E) The labeled structure is a branch of the thyrocervical trunk.

Neck

1. Which of the following statements holds true for the structure labeled with number 1?

Sternocleidomastoideus muscle

(A) This structure forms the medial border of the medial triangle of the neck and the lateral border of the lateral triangle of the neck.

(B) This structure is innervated by the facial nerve and the cervical plexus.

(C) This structure forms the medial border of the carotid triangle.

(D) With the head fixed, this structure functions as an accessory respiratory muscle.

(E) This structure is innervated by the vagus nerve and the cervical plexus.

2. Which of the following statements for the structure labeled with number 2 is wrong?

Sympathetic trunk

(A) The labeled structure stimulates the right ventricle.

(B) Parts of the labeled structure are the superior and middle cervical ganglion.

(C) Part of the labeled structure is the stellate ganglion.

(D) The labeled structure is comprised of 22-23 paravertebral ganglia.

(E) The labeled structure is comprised of 50 autonomous ganglia.

3. Which of the following statements holds true for the structure labeled with number 3?

Deep cervical ansa

(A) The labeled structure innervates the suprahyoid musculature motorically.

(B) The labeled structure innervates the suprahyoid musculature sensorial.

(C) The labeled structure innervates the platysma.

(D) The labeled structure accompanies the phrenic nerve.

(E) The labeled structure is comprised of nerve branches from the spinal segments C1-C3.

4. Which of the following statements holds true for the structure labeled with number 4?

Vagus nerve

(A) Branches of the labeled structure are the superior and recurrent laryngeal nerves.

(B) The labeled structure takes a course along the anterior scalene muscle.

(C) The labeled structure takes a course laterally outside of the carotid sheath.

(D) The labeled structure takes a course along the middle scalene muscle.

(E) The labeled structure exchanges branches with the vestibulocochlear nerve.

5. Which of the following statements holds true for the structure labeled with number 5?

<u>External carotid artery</u>

(A) The labeled structure is located anterior to the common carotid artery.

(B) The labeled structure is located anterior to the internal carotid artery.

(C) The labeled structure splits into three terminal branches: the maxillary artery, superficial temporal artery and facial artery.

(D) The labeled structure splits into three terminal branches: the maxillary artery and the profound temporal artery.

(E) The labeled structure is crossed over by the glossopharyngeal nerve and crossed under by the hypoglossal nerve.

6. Which of the following statements holds true for the structure labeled with number 6?

<u>Common carotid artery</u>

(A) The labeled structure originates directly from the aortic arch on the specimen’s right side.

(B) A strong compression over a longer period of time of the labeled structure in the area of the carotid sinus can lead to a cardiac arrest.

(C) The branches of the labeled structure cross under the sternocleidomastoid muscle in medial direction.

(D) The labeled structure continues cranial to the posterior belly of the digastric muscle as the brachiocephalic trunk.

(E) The labeled structure and its branches form the main vascular supply for the lateral region of the neck.

7. Which of the following statements holds true for the structure labeled with number 7?

Internal jugular vein

(A) The labeled structure is one of the most important epifascial vessels in the lateral region of the neck.

(B) The labeled structure does not give off or take in any branches in the cervical region.

(C) The labeled structure is the most lateral structure of the lateral triangle of the neck.

(D) The labeled structure is via the superior thyroid vein responsible for the venous drainage of the superior portion of the thyroid gland.

(E) The labeled structure shows a direct positional relationship to the nerve point of the neck.

8. Which of the following statements holds true for the structure labeled with number 8?

Phrenic nerve

(A) The labeled structure innervates the infrahyoid musculature.

(B) The labeled structure takes course along the anterior scalene muscle and between the subclavian artery and vein.

(C) The labeled structure is responsible for the parasympathetic innervation of the thyroid gland.

(D) The labeled structure is a branch of the brachial plexus.

(E) The labeled structure takes a course inside the middle fascia of the neck along the inner surface of the sternocleidomastoid muscle.

9. Which of the following statements holds true for the structure labeled with number 9?

<u>**Mylohyoid muscle**</u>

(A) The labeled structure is innervated by the superior laryngeal nerve.

(B) The labeled structure is located cranially between the hyoid bone and the mandible.

(C) Together with the tongue musculature the labeled structure forms the base of the tongue.

(D) The labeled structure is responsible for bringing down the hyoid bone.

(E) The labeled structure is connected with the cervical viscera via the white line of the neck.

10. Which of the following statements holds true for the structure labeled with number 10?

(A) The labeled structure is connected with the hyoid bone via the cricothyroid ligament.

(B) The elastic conus of the labeled structure is more prominent in males than in females and forms the so-called Adams apple.

(C) The ligament cranial to the labeled structure is pierced by the superior laryngeal nerve.

(D) The thyroid gland is located cranially to the labeled structure.

(E) When there is danger of asphyxia the ligament cranial to the labeled structure can be cut to perform a coniotomy.
